# Trends in Insurance Coverage for Acupuncture, 2010-2019

**DOI:** 10.1001/jamanetworkopen.2021.42509

**Published:** 2022-01-12

**Authors:** Molly Candon, Arya Nielsen, Jeffery A. Dusek

**Affiliations:** 1 Department of Psychiatry, Perelman School of Medicine, The University of Pennsylvania, Philadelphia; 2 Department of Health Care Management, The Wharton School, The University of Pennsylvania, Philadelphia; 3 Leonard Davis Institute of Health Economics, University of Pennsylvania, Philadelphia; 4 Department of Family Medicine and Community Health, Icahn School of Medicine at Mount Sinai, New York, New York; 5 University Hospitals Connor Whole Health, Cleveland Medical Center, Cleveland, Ohio; 6 Department of Family Medicine and Community Health, Case Western Reserve University, Cleveland, Ohio

## Abstract

This survey study examines US trends in insurance coverage for acupuncture from 2010 through 2019.

## Introduction

Acupuncture is recommended as part of comprehensive pain care for low back pain, neck pain, and fibromyalgia by agencies including the Agency for Healthcare Research and Quality.^1^ Additional evidence indicates that complementary and alternative medicine, including acupuncture, is associated with reductions in total health care spending among patients with chronic back pain.^2^

Research suggests that insurance coverage for acupuncture is inconsistent, although there is a lack of published data concerning coverage in most states.^3^ One survey of 45 commercial, Medicaid, and Medicare Advantage (ie, Part C) health plans found that only one-third of plans covered acupuncture, suggesting most patients pay for acupuncture entirely out of pocket.^4^ When insurers covered acupuncture, cost sharing was higher than other nonpharmacological interventions, and insurers tended to cover few indications and clinician types.^3,4^

Here, we document trends in insurance coverage for acupuncturist visits using a nationally representative survey. Given Medicare Part B’s 2020 decision to reimburse acupuncture for low back pain, we hypothesized that insurance coverage increased over time.^3^

## Methods

We examined insurance coverage for acupuncturist visits between 2010 and 2019 using the Medical Expenditure Panel Survey (MEPS).^5^ The MEPS data are publicly available and are collected using sampling methods consistent with the American Association for Public Opinion Research (AAPOR) reporting guideline. Respondents provided verbal consent to participate in the MEPS. Acupuncture use and demographic characteristics, including race and ethnicity, were self-reported. If not ascertained in the MEPS or National Health Interview Survey, race and ethnicity were assigned based on the respondent’s relationship to other household members. Race and ethnicity categories included Hispanic, non-Hispanic Asian, non-Hispanic Black, non-Hispanic White, and non-Hispanic other race or multiple races. This survey study was considered exempt by the Institutional Review Board at the University of Pennsylvania, Philadelphia.

We measured the share of respondents 18 years or older with at least 1 acupuncturist visit. Among acupuncture users, we calculated the (1) total annual amount paid for acupuncturist visits, (2) annual amount paid out of pocket for acupuncturist visits, (3) share of acupuncturist visits with any insurance coverage, and (4) percentage paid out of pocket for acupuncturist visits. We calculated the outcomes in 2-year intervals to improve precision and compared outcomes in 2010-2011 vs 2018-2019. Statistical significance was defined as a CI excluding 0. Analyses were performed in Stata, version 17.0 (StataCorp LLC).

## Results

The overall full-year file MEPS response rate varied from 39.5% in 2019 to 56.3% in 2012 during the study period. (An accessible table with the original number of surveys distributed is not available given the National Health Interview Survey and MEPS logistics.) The proportion of respondents with at lease 1 acupuncturist visit increased from 0.4% in 2010 to 0.8% in 2019 (Figure). Of the 1344 acupuncture users, 922 (68.6%) were female, 251 (18.7%) were Asian, 85 (6.3%) were Black, 201 (15.0%) were Hispanic, and 768 (57.1%) were White. Respondents had a mean (SD) age of 51.9 (14.9) years.

**Figure.**
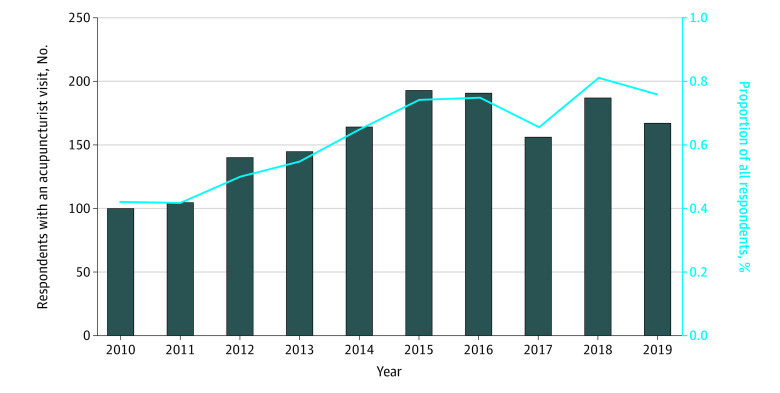
Trends in Office Visits With Acupuncturists in the Medical Expenditure Panel Survey, 2010-2019 Between 2010 and 2019, 1344 respondents reported at least 1 visit with an acupuncturist.

Spending on acupuncturist visits increased during the study period (Table). The total annual amount paid for acupuncturist visits was a mean of $593.00 (95% CI, $460.29-$725.70) in 2010-2011 and $1021.57 (95% CI, $806.32-$1236.82) in 2018-2019; the increase was statistically significant (mean difference, $429; 95% CI, $129-$728). While the annual amount paid out of pocket increased from a mean of $375.51 (95% CI, $286.12-$464.90) in 2010-2011 to $554.26 (95% CI, $383.54-$724.99) in 2018-2019, the change was not statistically significant (mean difference, $179; 95% CI, $−55 to $412). The increase in spending was largely accounted for by an increase in acupuncturist visits among users, which increased from a mean of 5.4 (95% CI, 4.2-6.6) visits per person in 2010 to 8.2 (95% CI, 6.4-9.9) visits per person in 2019.

**Table.** Trends in Spending and Insurance Coverage for Acupuncturist Visits in the Medical Expenditure Panel Survey, 2010-2019^a^

Year	No. of unique visits with an acupuncturist^b^	Mean (95% CI)			
		Annual amount paid for acupuncturist visits, $^c^		Share of acupuncturist visits with any insurance coverage, %	Proportion paid out of pocket for acupuncturist visits, %
		Total	Out of pocket		
2010-2011	1052	593.00 (460.29-725.70)	375.51 (286.12-464.90)	41.1 (38.1-44.0)	66.9 (64.4-69.3)
2012-2013	1665	588.45 (454.96-721.95)	286.54 (228.34-344.74)	43.9 (41.5-46.3)	58.2 (55.9-60.4)
2014-2015	2090	583.58 (494.66-672.50)	314.27 (254.86-373.69)	43.4 (41.3-45.5)	61.0 (59.2-62.9)
2016-2017	2205	728.31 (593.47-863.16)	358.58 (276.28-440.89)	47.8 (45.7-49.9)	57.6 (55.7-59.4)
2018-2019	2656	1021.57 (806.32-1236.82)	554.26 (383.54-724.99)	50.2 (48.3-52.1)	57.5 (55.8-59.1)

^a^ Data were adapted from Blewett et al.^5^

^b^ Visits with negative spending amounts were excluded.

^c^ Dollar amounts were adjusted for inflation to 2019 dollars using the health care consumer price index.

The share of acupuncturist visits with any insurance coverage increased from a mean of 41.1% (95% CI, 38.1%-44.0%) in 2010-2011 to 50.2% (95% CI, 48.3%-52.1%) in 2018-2019 (mean difference, 9.1 percentage points ; 95% CI, 5.6-12.7 percentage points), while the percentage paid out of pocket declined from a mean of 66.9% (95% CI, 64.4%-69.3%) in 2010-2011 to 57.5% (95% CI, 55.8%-59.1%) in 2018-2019 (mean difference, −9.4 percentage points; 95% CI, −12.5 to −6.3 percentage points).

## Discussion

Between 2010 and 2019, the share of MEPS respondents who had at least 1 acupuncturist visit increased from 0.4% to nearly 0.8%, and the rate of insurance coverage increased by 9.1 percentage points. This finding aligns with a recent study from Oregon Medicaid, which found both expanded coverage for acupuncture and more acupuncture utilization.^6^

Nevertheless, half of respondents reported no insurance coverage for acupuncturist visits in 2018-2019, and most spending occurred out of pocket. Insurers have cited an inconsistent evidence base as a reason behind coverage decisions, yet acupuncture has been shown to be effective for various pain conditions that are not always covered.^1,3,4^ For example, Medicare reimburses acupuncture for low back pain, excluding other conditions for which acupuncture is recommended.^1,3^

The study had notable limitations. The MEPS is nationally representative, but its size resulted in fewer than 1400 respondents with an acupuncturist visit. Measures were self-reported. We cannot assess whether respondents were referred to acupuncturists, nor did we examine indications associated with acupuncture therapy.

In conclusion, the results of this study indicate that insurance coverage for acupuncturist visits has increased, but most costs are paid out of pocket. Insurers should be encouraged to cover safe, low-cost, and evidence-based approaches to comprehensive pain care, including acupuncture therapy.
